# On the Effect of Thermodynamic Equilibrium on the Assembly Efficiency of Complex Multi-Layered Virus-Like Particles (VLP): the Case of Rotavirus VLP

**DOI:** 10.1371/journal.pcbi.1002367

**Published:** 2012-02-16

**Authors:** António Roldão, Maria Candida M. Mellado, J. C. Lima, Manuel J. T. Carrondo, Paula M. Alves, R. Oliveira

**Affiliations:** 1 Instituto de Tecnologia Química e Biológica-Universidade Nova de Lisboa (ITQB-UNL), Oeiras, Portugal; 2 Instituto de Biologia Experimental e Tecnológica (IBET), Oeiras, Portugal; 3 REQUIMTE, Faculdade de Ciências e Tecnologia/Universidade Nova de Lisboa (FCT/UNL), Caparica, Portugal; 4 Faculdade de Ciências e Tecnologia/Universidade Nova de Lisboa (FCT/UNL), Monte de Caparica, Portugal; Iowa State, United States of America

## Abstract

Previous studies have reported the production of malformed virus-like-particles (VLP) in recombinant host systems. Here we computationally investigate the case of a large triple-layered rotavirus VLP (RLP). In vitro assembly, disassembly and reassembly data provides strong evidence of microscopic reversibility of RLP assembly. Light scattering experimental data also evidences a slow and reversible assembly untypical of kinetic traps, thus further strengthening the fidelity of a thermodynamically controlled assembly. *In silico* analysis further reveals that under favourable conditions particles distribution is dominated by structural subunits and completely built icosahedra, while other intermediates are present only at residual concentrations. Except for harshly unfavourable conditions, assembly yield is maximised when proteins are provided in the same VLP protein mass composition. The assembly yield decreases abruptly due to thermodynamic equilibrium when the VLP protein mass composition is not obeyed. The latter effect is more pronounced the higher the Gibbs free energy of subunit association is and the more complex the particle is. Overall this study shows that the correct formation of complex multi-layered VLPs is restricted to a narrow range of association energies and protein concentrations, thus the choice of the host system is critical for successful assembly. Likewise, the dynamic control of intracellular protein expression rates becomes very important to minimize wasted proteins.

## Author Summary

Virus-like particles (VLP) are multi-protein structures with potential to be used in biomedicine as therapeutic or prophylactic vaccines against many worldwide diseases. However, due to their complex process of assembly, production yields are commonly low hindering a fast transition from lab- to industrial-scale and ultimately to the patient. It is thus vital to understand the assembly mechanisms of these particles and to apply rational-oriented strategies for the manipulation of the host system and/or process conditions. In this context, mathematical models built on stability, kinetic and thermodynamic principles of protein macrostructures and respective assembly pathways are key elements. Indeed, using mathematical and thermodynamic concepts as building blocks, the model herein postulated provides valuable insights about the feasibility of VLP production systems. It demonstrates that the formation of complete and theoretically biological active VLP is restricted to a narrow range of volatile macro and micro physicochemical environments as well as intracellular protein expression rates. This study also finds direct application in the design of optimal *in vitro* physicochemical conditions for disassembly-reassembly experiments towards economical VLP production.

## Introduction

The *in vivo* assembly of virus-like particles (VLP), multi-protein structures, in unnatural host systems results many times in predominantly malformed structures because the host biology provides neither the adequate spatial and temporal protein expression conditions nor the ideal thermodynamic environment for their assembly into VLP. A prominent example is the prophylactic human papillomavirus (HPV)-VLP vaccine developed by Merck & Co., Inc. (Gardasil™). In this case, *in vitro* disassembly and reassembly operations were essential for economical production of the correctly assembled VLP from the L1 proteins of HPV types 6, 11, 16 and 18 expressed in *Saccharomyces cerevisiae*. Understanding the control of VLP assembly is vital in order to adequately engineer host systems and to study the feasibility of controlled *in vitro* assembly. In this context, mathematical models built on stability, kinetic and thermodynamic principles of protein macrostructures and respective assembly pathways are key elements to better understand and design VLP assembly control strategies.

The self-assembly of virus capsids has been extensively studied by Zlotnick and co-authors [1]–[3]. The problem of capsid assembly was initially addressed as a cascade of low-order reactions with driving force the thermodynamic-equilibrium relationship between monomers and complete capsids [2]. This formalism uses two basic parameters to control virus capsid assembly, namely the concentration of structural subunits and the Gibbs free energy of subunit association (**ΔG^0^_n_**). To handle kinetically trapped products, a kinetically limiting (KL) model was afterwards developed [1]. This model stems from the equilibrium model [2], which was modified to consider the existence of rate-limiting steps. Kinetic traps occur when assembly is initiated too many times, leaving an insufficient concentration of free subunits to allow the reaction to proceed to completion. For instance, if the association energy in the initial assembly steps are much higher than that of subsequent steps, fast depletion of free subunits occurs which leads to the accumulation of stable intermediates and subsequently to kinetically trapped products. This phenomenon can be also observed at very high protein concentrations [4]. The KL model has been applied to analyse the assembly of many virus capsids and to derive techniques for estimating reaction parameters from kinetic data [5]–[9]. More recently, a landscape representation for the complex association reaction of virus capsids has been proposed [3]. The representation indicates that few of the many possible intermediates make a significant (measurable) contribution to assembly, consistent with earlier descriptions of thermodynamically independent pathways [2], [10].

Several other in general more complex modelling strategies were proposed to describe viral capsid assembly based on molecular dynamics [11], thermodynamics as self-organization of discs on a sphere [12], combinatorial optimization studies [10], “local rules” approach [13], stochastic icosahedral capsid growth [14] and protein aggregation by nucleated polymerization [15]. In Hagan and Chandler 2006, a class of capsomer models was created to simulate the assembly of capsid-like objects from subunits of different geometries using Newtonian dynamics [16]. Others have used the viral tiling theory to describe the assembly of structural subunits formed from pentamers that differ by the structure of the inter-subunit bonds that surround them [17]. They combine the information on local environments around pentamers with the thermodynamic-equilibrium model of Zlotnick [2]. The major drawback in viral tiling theory is that distinct configurations may be energetically preferred when attaching a further building block. This gives rise to a tree of assembly pathways rather than a single assembly pathway as in Zlotnick's framework, significantly complicating the assembly process. With varying complexity, the vast majority of modelling studies in the literature addressed single-layered virus capsid assembly only. Here we explore the production of more complex multi-layered particles, namely triple-layered rotavirus VLP (RLP) produced in the baculovirus/insect cell (B/IC) expression system. RLP are spherical shaped particles composed by three viral proteins (vp) of rotavirus arranged in a triple layered structure: vp2 - 102.7 kDa (innermost layer) [18]; vp6 - 44.9 kDa (middle shell) [19]; vp7 - 37.2 kDa (outer layer) [19]. Experimental data shows that correctly assembled particles account for only 12% of the total mass of proteins expressed [20]. Unfortunately, few experimentally supported information exists of intra- and inter-protein binding energies, rates and orders for assembly reactions that could enlighten the causes of particle malformation. A recent study of *in vitro* assembly/disassembly of RLPs explored the effect of physicochemical parameters on RLPs stability [21]. This study showed that protein macrostructures are highly dependent on the pH, ionic strength and temperature. To better understand the causes of particle malformation we have developed an RLP assembly mathematical model based on well-known principles (template-driven assembly, hierarchical assembly and Zlotnick's thermodynamic-equilibrium framework [2]), analysed *in vitro* assembly and disassembly experimental data and then computationally explored the factors that control RLP assembly.

## Results/Discussion

### Evidence from in vitro assembly experiments

RLPs are a potentially relevant vaccine candidate against rotavirus disease. The production process of such complex particles is however far from being efficient, hindering its transition from lab- to large-scale production. Indeed, experimental data shows that contaminants such as double-layered particles composed by vp2 and vp6 (DLPs) (vp7 is easily “peeled off” from particles outer layer [22]) account for almost 88% of the total mass of proteins expressed [20]. In order to increase process yields and product quality, it is critical to understand how these particles assemble and behave at specific physico-chemical macro-environments. Since *in vivo* testing is highly complex and in most cases inaccurate, involving complicated networks of molecular interactions and different cell compartments, the *in vitro* analysis of RLPs assembly from DLPs and purified vp7 emerge as the most suitable and simple alternative for clarifying how the macro-environment impacts on the physical characteristics of these particles.

The assembly of the outer vp7 layer can be described by the lumped equilibrium reaction
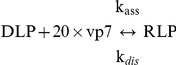
with **k_ass_** and **k_dis_** the reaction rate constants of RLP assembly and disassembly, respectively. The ratio of the reaction rate constants is the equilibrium constant, **K_eq_**:
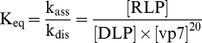
(1)The Gibbs free energy of vp7 layer assembly on top of DLPs, **ΔG^0^_layer,vp7_**, can be defined as:
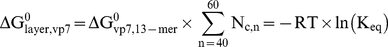
(2)Based on Eq. 2, the equilibrium constant **K_eq_** is related with the Gibbs free energy of vp7 subunit association, **ΔG^0^_vp7,13-mer_**, by:
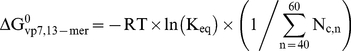
(3)The Gibbs free energy of vp7 subunit association for the *in vitro* RLPs assembly experiments was calculated using the formula (see Table S1):
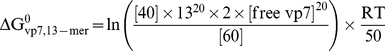
(4)where **[40]** is the concentration of DLP at equilibrium, **[DLP]_e_**, [*free* vp7] is the concentration of unassociated vp7 subunits at equilibrium, **[svp7]_e_** and **[60]** is the concentration of RLP at equilibrium, **[RLP]_e_**. The obtained results are summarized in table 1.

**Table 1:** *In vitro* assembly and disassembly of RLPs.

Assembly	Assembly condition	Ass_eff_	Temperature (K)	ΔG^0^_vp7,13-mer_ (cal.mol^−1^)	ΔG^0^_layer,vp7_ (kcal.mol^−1^)
	pH 5.5/[NaCl] = 0.1 M/[Ca^2+^] = 1 mM	0.48	298.15	−4830	−242
	pH 8.0/[NaCl] = 0.1 M/[Ca^2+^] = 1 mM	<0.10	298.15	>−4675	>−234
	pH 5.5/[NaCl] = 0.5 M/[Ca^2+^] = 1 mM				
	pH 5.5/[NaCl] = 0.1 M/[Ca^2+^] = 5 mM				
	pH 5.5/[NaCl] = 0.1 M/[Ca^2+^] = 1 mM	<0.10	308.15	>−4832	>−242

Disassembly	Disassembly condition		Dis_eff_	Temperature (K)	ΔG^0^_vp7,13-mer_ (cal.mol^−1^)	ΔG^0^_layer,vp7_ (kcal.mol^−1^)
	TNC buffer	EGTA 1 mM	0.72	308.15	−3298	−165
		EDTA 1 mM	0.72		−3297	−165
		EGTA 2 mM	0.22		−3609	−180
		EDTA 2 mM	0.19		−3655	−183
	D-PBS buffer	EGTA 1 mM	0.66	308.15	−3320	−166
		EDTA 1 mM	0.46		−3420	−171
		EGTA 2 mM	0.33		−3507	−175
		EDTA 2 mM	0.99		−3175	−159

Reassembly	Disassembly condition	Reassembly condition	Ass_eff_	Temperature (K)	ΔG^0^_vp7,13-mer_ (cal.mol^−1^)	ΔG^0^_layer,vp7_ (kcal.mol^−1^)
	EGTA 2 mM (TNC)	[Ca^2+^] = 5 mM and T = 25°C	0.47	298.15	−3626	−181
	EDTA 2 mM (TNC)		0.28		−3586	−179
	EGTA 2 mM (D-PBS)		0.54		−3570	−179
	EDTA 2 mM (D-PBS)		0.80		−3524	−176

Results show that the increase in pH, concentration of sodium chloride ([NaCl]) or concentration of calcium ([Ca^2+^]) had a negative impact on the Gibbs free energy of vp7 subunit association; the **ΔG^0^_vp7,13-mer_** values are higher than the one estimated for the standard condition of assembly (pH 5.5, 25°C, 0.1 M of NaCl and 1 mM of calcium ion (Ca^2+^)). This indicates that the equilibrium constants for the assembly reactions at pH 8, [NaCl] = 0.5 M or [Ca^2+^] = 5 mM decreased and, if **K_eq_** decreased, product formation became less favoured; hence, the assembly efficiencies are lower.

The dependency between temperature **T** and the equilibrium constant **K_eq_** is described by the van't Hoff equation:
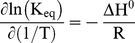
(5)where **ΔH^0^** is the standard enthalpy of reaction (cal.mol^−1^). The **ΔH^0^** and the standard entropy of reaction, **ΔS^0^** (cal.mol^−1^.K^−1^), can be calculated by plotting the natural logarithm of the equilibrium constant versus the reciprocal temperature as follows:
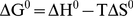
(6)

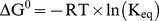
(7)

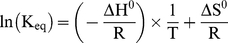
(8)with the slope equal to (−ΔH^0^/R) and the intercept equal to (−ΔS^0^/R).

The *in vitro* assembly experiments carried out at 25°C and 35°C show that the increase in temperature reduced significantly the efficiency of RLP assembly (table 1). For this particular case of vp7 association to the DLP to form RLP and in this narrow temperature range where the enthalpy term can be considered constant, these results imply that the increase in temperature turned the net entropy term more positive (−TΔS^0^) and subsequently the ΔG^0^. This means that ΔS^0^ for the association must be negative and therefore results from electrostatics interactions and hydrogen bonds (oriented) that weaken with temperature and not from hydrophobic interactions (favoured by temperature) where the ΔS^0^ is normally positive due to the release of molecules of solvent from the contact region between proteins. These results corroborate previous findings showing that the interaction vp6–vp7 is mainly due to van der Waals contacts and hydrogen bonds [23], and our own experimental data showing that the stability of RLP is highly dependent on the pH and Ca^2+^ concentration (see above).

### Evidence from in vitro disassembly experiments

*In vitro* disassembly experiments were performed at different concentrations of ethylene glycol tetraacetic acid (EGTA) and ethylene diamine tetraacetic acid (EDTA) (see table 1). For each experiment, the Gibbs free energy of vp7 subunit association was calculated based on Eq. 4; the obtained results are summarized in table 1.

It is clear from Eqs. 1 to 3 that the equilibrium constant **K_eq_** is inversely proportional to the Gibbs free energy of vp7 subunit association and to the disassembly efficiency (Dis_eff_); the lower the **K_eq_**, the higher is the **ΔG^0^_vp7,13-mer_** and the disassembly efficiency. Therefore, the optimal condition to disassemble RLP is to use EDTA 2 mM in Dulbecco's phosphate buffered saline (D-PBS) as it presents the highest **ΔG^0^_vp7,13-mer_** (table 1). Interestingly, the concentration of chelating agent appears to be correlated with the **ΔG^0^_vp7,13-mer_**; higher concentrations correspond to lower **ΔG^0^_vp7,13-mer_**. The exception is the case of EDTA 2 mM in D-PBS where **ΔG^0^_vp7,13-mer_** is higher than the one achieved at EDTA 1 mM.

Upon the disassembly of RLPs with EDTA and EGTA 2 mM, reassembly was performed with dialysis against buffer with calcium 5 mM and decreasing the temperature to 25°C (table 1). As a rule, the **ΔG^0^_vp7,13-mer_** values for the reassembly experiments are lower than the ones obtained for the disassembly. This happens because lower temperatures improve RLP assembly efficiency (Ass_eff_) as demonstrated above (see previous section). Moreover, by increasing the concentration of calcium, the vp7 attachment to DLP is increased. However, very high concentrations of calcium (5 mM; see table 1) seem to have a deleterious effect on the association. Summing up, the combined effect of optimal calcium concentration and temperature resulted in the increase in **K_eq_** and corresponding decrease in **ΔG^0^_vp7,13-mer_**.

### Evidence from light scattering data

**Figure 1:**
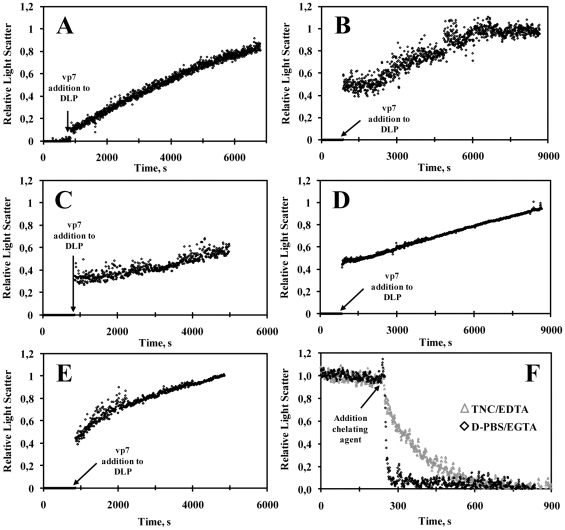
Kinetics of *in vitro* RLP assembly and disassembly by 90° light scattering. Transition of DLP to RLP under standard condition (TNC buffer at pH 5.5, 25°C, NaCl 0.1 M and Ca^2+^ 1 mM) (graph A) and upon increase in pH (8 – graph B), Ca^2+^ (5 mM – graph C), temperature (35°C – graph D) and NaCl (0.5 M – graph E). Dissociation of RLP to DLP at 25°C in TNC with 1 mM of EDTA (grey triangle) or in D-PBS with 1 mM of EGTA (black diamond) (graph F). The arrows indicate the times at which vp7 was added to DLP (graphs A to E) or the addition of chelating agents (graph F).

The postulated thermodynamic-equilibrium model is not applicable if assembly is based on kinetically trapped products rather than products and intermediates approaching equilibrium. In order to screen for possible kinetic trap formation, the kinetics of *in vitro* RLP assembly and disassembly were monitored by light scattering spectroscopy for a selected set of conditions (see figure 1). A slow and monotonic increase in relative light scatter from 0 (DLP) to 1 (RLP) was observed in all in vitro disassembly experiments (figures 1a–e). The initial rate is steep in most cases, the maximum signal intensity is constantly increasing and the slope of the final phase is around 4–9×10^−3^ M^−1^ s^−1^ (see calculation method in [21]). These dynamics are untypical of kinetic traps. In the case of kinetic traps, the maximum light scatter signal attenuates after the fast initial rate, indicative of intermediate species accumulation to high concentrations and depletion of structural subunits [1].

In the RLP disassembly experiments an exponential-like decrease in relative light scatter from 1 (RLP) to 0 (DLP) was observed (figure 1f). Upon the action of chelating agents, RLPs dissociate and disassembled structural subunits (vp7) and icosahedra (DLP) establish equilibrium. This complete transition from RLP to DLP is consistent with the principle of microscopic reversibility. Noteworthy is the time needed for disassembly reactions to approach equilibrium, less than 900 s, significantly lower than that needed for RLP assembly (figures 1a–e). These data strongly suggest that RLPs are held together by many weak interactions, similar to what occurs with many viruses [9]. Thus, once the first subunit is removed from the metastable complete RLP, neighboring subunits have fewer bonds and further disassembly is rapid. Diffusional and affinity level limitations can also support the observed behavior [21]. Although the existence of kinetic traps cannot be completely ruled out, the collected evidence of slow and reversible assembly decreases the probability of kinetic traps and increases the fidelity of assembly due to thermodynamics [8]–[9].

### Simulation of vp2 singe-layer assembly

**Figure 2:**
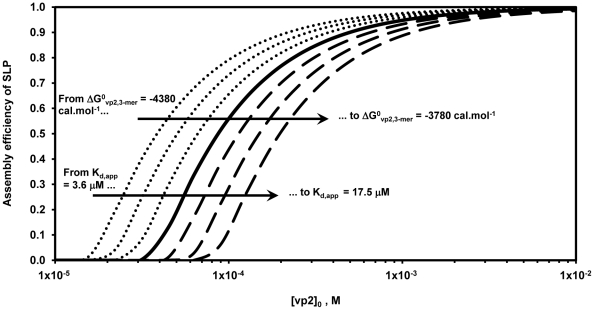
SLP assembly efficiency as function of the initial vp2 concentration, [vp2]_0_, for different Gibbs free energy of vp2 subunit association values, ΔG^0^_vp2,3-mer_. The full line represents **ΔG^0^_vp2,3-mer_** = −4.08 kcal.mol^−1^ as estimated by Zlotnick 1994, the dash lines represent **ΔG^0^_vp2,3-mer_**>−4.08 kcal.mol^−1^ and the dot lines represent **ΔG^0^_vp2,3-mer_**<−4.08 kcal.mol^−1^. **K_d,app_** represents the pseudo-critical concentration that satisfies the constraint **[1]** (concentration of unassociated vp2 subunits at equilibrium - **[1]** = **[svp2]_e_**) = **[20]** (complete SLP) = **K_d,app_** at equilibrium for a specific **ΔG^0^_vp2,3-mer_**.

For simulation of the innermost vp2 layer assembly, Zlotnick's equilibrium model was simulated according to the parameters specified in Table S2. Figure 2 shows the simulated assembly efficiency as function of the initial vp2 concentration, **[vp2]_0_**, for different values of Gibbs free energy of vp2 subunit association (**ΔG^0^_vp2,3-mer_**). It is important to highlight that the following analysis is only valid under the range of Gibbs free energy tested (from −4.38 to −3.78 kcal.mol^−1^), where thermodynamic control of assembly prevails over kinetic traps. Outside these boundaries and especially for very negative Gibbs free energies, the dissociation rate becomes negligible and the thermodynamic control is no longer in play, i.e. the postulated thermodynamic model is no longer valid as it does not take into account kinetic trapping.

For the range of Gibbs free energy tested (from −4.38 to −3.78 kcal.mol^−1^), the assembly efficiency curves follow a sigmoidal shape with respect to **[vp2]_0_**. Independently of **ΔG^0^_vp2,3-mer_**, few particles are formed at very low **[vp2]_0_**; above a given critical **[vp2]_0_** value, most subunits assemble into macrostructures. This threshold represents the minimum initial concentration of vp2 needed to ensure a measurable single-layered vp2 particles (SLP) formation, i.e., the pseudo-critical concentration, **K_d,app_**, that satisfies the constraint **[1]** = **[20]** = **K_d,app_** at equilibrium for a given **ΔG^0^_vp2,3-mer_**. The **K_d,app_** can be derived from Table S2:
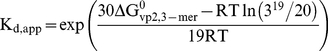
(9)It is clear that **K_d,app_** increases with **ΔG^0^_vp2,3-mer_**. For instance, **K_d,app_** = 3.6 µM for **ΔG^0^_vp2,3-mer_** = −4.38 kcal.mol^−1^ whereas at **ΔG^0^_vp2,3-mer_** = −3.78 kcal.mol^−1^ **K_d,app_** increases almost 4-fold to 17.5 µM. These results are in agreement with the principle of the Gibbs free energy – “every system seeks to achieve a minimum of free energy” – i.e. the more negative is the **ΔG^0^**, the higher is the equilibrium constant (see Eq. 7) and subsequently the lower is the concentration of **[vp2]_0_** necessary to maximize SLP assembly. The **[vp2]_0_** window for transition of unassembled structural subunits to intact icosahedra at **ΔG^0^_vp2,3-mer_** = −4.08 kcal.mol^−1^ is 0.03–6 mM, a significantly broader transition window than that reported for higher **ΔG^0^_vp2,3-mer_** values (more positive). This clearly demonstrates that low **ΔG^0^_vp2,3-mer_** corresponds to a better macro-environment for the assembly of vp2 subunits into SLP than high **ΔG^0^_vp2,3-mer_**. In other words, at initial vp2 concentration not favourable for maximum SLP assembly (e.g. 0.03 mM), the lower the **ΔG^0^_vp2,3-mer_**, the higher is the equilibrium constant and subsequently the higher is the assembly efficiency.

The aforementioned pseudo-critical concentration **K_d,app_** can be also interpreted as the dissociation constant for a single contact in the final structure when the concentration of species **1** and **20** coincide [2]. As shown in figure 2, decreasing **ΔG^0^_vp2,3-mer_** also decreases the **K_d,app_**, suggesting that the reaction **SLP→20×vp2** is not favoured. In other words, the more negative is **ΔG^0^_vp2,3-mer_** the higher is the association constant for a subunit-subunit interaction, **K_contact_** [24]:
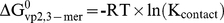
(10)Since the overall SLP assembly constant, **K_layer_**, is proportional to **K_contact_** [24]:
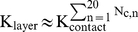
(11)an increase in **K_contact_** favours the reaction **20×vp2→SLP**, increasing species **20** formation and consequently the assembly efficiency. Assuming a thermodynamic control of the assembly, Eqs. 10 and 11 clearly demonstrate that as more contacts are established *per* subunit, the lower is **K_d,app_** and the more stable is the particle formed.

### Simulation of complete triple-layered RLP assembly

For the simulation of the complete triple-layered RLP one needs to consider the equilibrium of the **n = 60** species according to parameters specified in Tables S1, S2, S3 and S4. The concentration of species **1** to **60** at equilibrium can be calculated as function of initial protein concentrations in solution before equilibrium. For given initial protein concentrations **[vp2]_0_**, **[vp6]_0_** and **[vp7]_0_**, the concentrations of intermediate species needs to be calculated recursively to ensure that protein material balances are obeyed, i.e. the initial amount of vp2, vp6 and vp7 must be equal to the sum of the amounts of proteins incorporated in all intermediate species. This translates into the following balance equations:
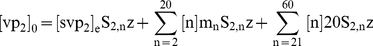
(12)

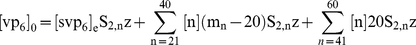
(13)

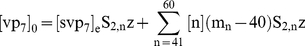
(14)where **[svp2]_e_**, **[svp6]_e_** and **[svp7]_e_** are the concentrations of unassociated vp2, vp6 and vp7 subunits at equilibrium, respectively, [**n**] the concentration of species **n** (calculated by Eq. 20), **m_n_** the number of structural subunits in species **n**, **S_2,n_** the degeneracy of the incoming subunit in species **n** and **z** the number of proteins in each oligomer. Parameters **S_2,n_** and **z** are specific for each layer: **S_2,n_** = 3 and **z** = 2 (vp2 layer); **S_2,n_** = 13 and **z** = 3 (vp6 and vp7 layers) (see above sections for details). This system can be easily solved by recursive calculation of the free **[svp2]_e_**, **[svp6]_e_** and **[svp7]_e_** until the material balance Eqs. 12 to 14 are obeyed to a given tolerance.

RLP assembly simulations were performed for three initial protein concentration scenarios:

- **[vp2]_0_** = 1.2 M, **[vp6]_0_** = 7.8 M and **[vp7]_0_** = 7.8 M;
- **[vp2]_0_** = 1.2 M, **[vp6]_0_** = 7.8 M and **[vp7]_0_** = 0.78 M;
- **[vp2]_0_** = 12 M, **[vp6]_0_** = 7.8 M and **[vp7]_0_** = 7.8 M.

**Figure 3:**
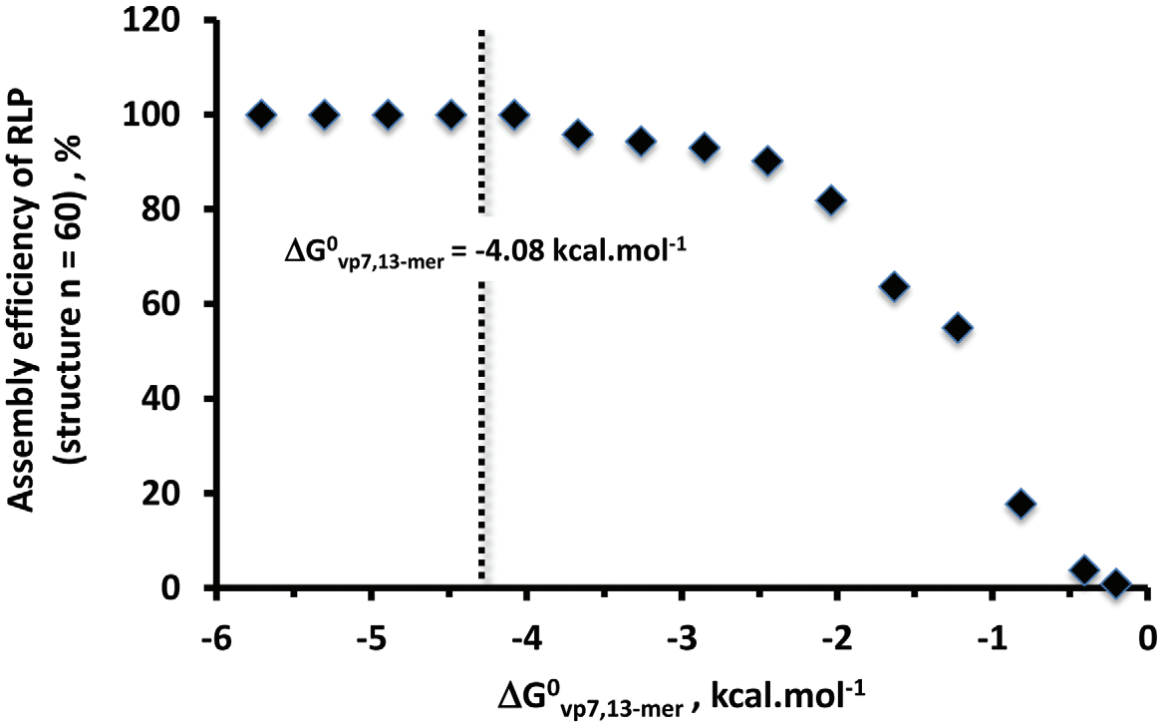
The effect of ΔG^0^_vp7,13-mer_ on RLP assembly efficiency when the initial protein concentration of vp2, vp6 and vp7 is 1.2 M, 7.8 M and 7.8 M, respectively, and the Gibbs free energy of vp2 and vp6 subunit association (ΔG^0^_vp2,3-mer_ and ΔG^0^_vp6,13-mer_, respectively) is equal to −4.08 kcal.mol^−1^. **ΔG^0^_vp7,13-mer_** varies between −4.08×[0.05 to 1.4] kcal.mol^−1^.

The rational for this choice is testing either protein limitation or excess in relation to the RLP stoichiometric composition (vp2∶vp6∶vp7 - 120∶780∶780 molecules). In this way, vp6 is provided at the exact stoichiometric ratio while scenarios of vp7 limitation and excess of vp2 are also explored. In order to avoid inaccurate assessment of the effect of these three different protein concentration scenarios on RLP assembly efficiency, it is critical to correctly define the Gibbs free energy of vp2, vp6 and vp7 subunit association (**ΔG^0^_vp2,3-mer_**, **ΔG^0^_vp6,13-mer_** and **ΔG^0^_vp7,13-mer_**, respectively). It should be noted that SLP and DLP assemble in vivo in different cell compartments than RLP (see Results and Discussion - section “*A model for rotavirus VLP assembly*“). It is thus rational to assume that **ΔG^0^_vp2,3-mer_**≈**ΔG^0^_vp6,13-mer_** while **ΔG^0^_vp7,13-mer_** may be quite different from the former. In addition, all **ΔG^0^** values must be coherent with experimental data. The values of **ΔG^0^_vp2,3-mer_** = **ΔG^0^_vp6,13-mer_** were fixed at −4.08 kcal.mol^−1^, assuming the same value of Hu and Bentley 2000 for the assembly of infectious bursal disease virus-VLPs and Zlotnick 1994 for the capsid assembly of spherical viruses. **ΔG^0^_vp7,13-mer_** was explored between −4.08×[0.05 to 1.4] kcal.mol^−1^ (Figure 3) in order to find the minimum value inducing near 100% assembly of structure n = 60 (complete RLP). By fine-tuning all **ΔG^0^** in this way, we guarantee that the concentration of structure **n** = 60 is maximized when stoichiometry (condition i) is satisfied. In this way, limitations in the thermodynamic control of RLP assembly that could jeopardize the analysis of modelling results are avoided. Simulations indicate that for **ΔG^0^_vp7,13-mer_**≤-4 kcal.mol^−1^ the assembly of structure n = 60 is maximized. .Based on this analysis, the Gibbs free energy of vp2, vp6 or vp7 subunit association was assumed equal to −4.08 kcal.mol^−1^ for all proteins.

**Figure 4:**
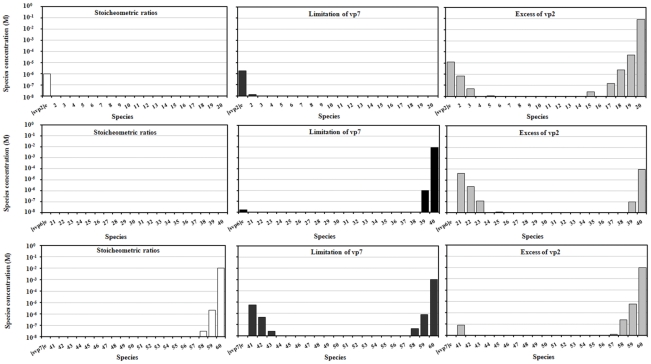
The concentration of assembly intermediates for three initial protein concentration scenarios: 1) **[vp2]_0_** = 1.2 M, **[vp6]_0_** = 7.8 M and **[vp7]_0_** = 7.8 M (stoichiometric ratios – white bars); 2) **[vp2]_0_** = 1.2 M, **[vp6]_0_** = 7.8 M and **[vp7]_0_** = 0.78 M (limitation of vp7 – black bars); 3) **[vp2]_0_** = 12 M, **[vp6]_0_** = 7.8 M and **[vp7]_0_** = 7.8 M (excess of vp2 – grey bars). The Gibbs free energy of vp2, vp6 and vp7 subunit association was assumed to be equal (**ΔG^0^_vp2,3-mer_** = **ΔG^0^_vp6,13-mer_** = **ΔG^0^_vp7,13-mer_** = −4.08 kcal.mol^−1^).

Figure 4 shows the simulated particles distribution at equilibrium for the three initial protein concentration scenarios, allowing to make the following observations:

- When initial protein concentrations (vp2, vp6 and vp7) obey the RLP stoichiometric ratio, the distribution of particles at equilibrium is dominated by a very small number of more stable structures, namely subunit **1** (i.e. **[1]** = **[svp2]_e_**) and structure **60** (complete RLP). The remaining intermediates are present but at residual concentrations. This shows that, for low Gibbs free energies, thermodynamics favours the formation of intact icosahedra whenever the respective proteins are not limiting.
- When vp7 is limiting, and according to Le Chatelier's principle, it should be expected that the equilibrium composition of the lumped reaction **DLP+20×vp7⇆RLP** shifts to the left with more DLP and less RLP formed. Indeed, a significant increase in the concentration of structure **40** (DLP) is observed along with a significant decrease of concentration of structure **60** (complete RLP). Concomitantly, moderate sized structures such as species **39**, **41** or **42** start to appear at significant concentrations. This clearly demonstrates that protein limitation negatively impacts particle assembly far beyond the stoichiometric ratio, since intermediate structures accumulate which represent unnecessary waste of proteins.
- An excess of vp2 concentration shifts the equilibrium of the lumped reaction **20×vp2⇆SLP** towards the generation of more SLP (Le Chatelier's principle). This equilibrium shift has also a profound impact on moderate sized structures such as species **17** to **23** or **39** to **41**; their concentrations increase significantly. The high concentrations observed for intermediate structures **20**, **17**–**23** and **39**–**41** indicate that the reaction **20×vp2⇆SLP+20×vp6⇆DLP+20×vp7⇆RLP** is not product-favoured. This demonstrates that proteins in excess have a profound impact in the equilibrium, which translates in the accumulation of a high number of intermediate species and low RLP assembly yield.

### Exploring the physicochemical RLP assembly design space

The assembly of protein complexes such as RLP can be optimized through the manipulation of viral protein concentrations and solution properties with impact on Gibbs free energy of subunit association (such as temperature, pH, ionic strength and particular ions concentration, namely Ca^2+^). In order to find these theoretical optimal conditions for RLP assembly, model simulations were performed for different values of Gibbs free energy of subunit association and vp6 and vp7 initial protein concentrations, **[vp6]_0_** and **[vp7]_0_**, respectively. The concentration of vp2, **[vp2]_0_**, was kept constant at 1 M. As in the previous section, the Gibbs free energies of vp2, vp6 and vp7 subunit association were assumed equal (**ΔG^0^_vp2,3-mer_** = **ΔG^0^_vp6,13-mer_** = **ΔG^0^_vp7,13-mer_**).

The RLP assembly efficiency is defined as the ratio between the mass of intact RLP at equilibrium and the total mass of initial proteins. To assess the effect of equilibrium, the assembly efficiencies derived from the thermodynamic model were compared to those obtained assuming an irreversible assembly reaction based on the RLP mass composition:
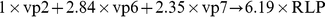
(15)In this irreversible assembly model, efficiency is determined uniquely by the concentration of proteins.

**Figure 5:**
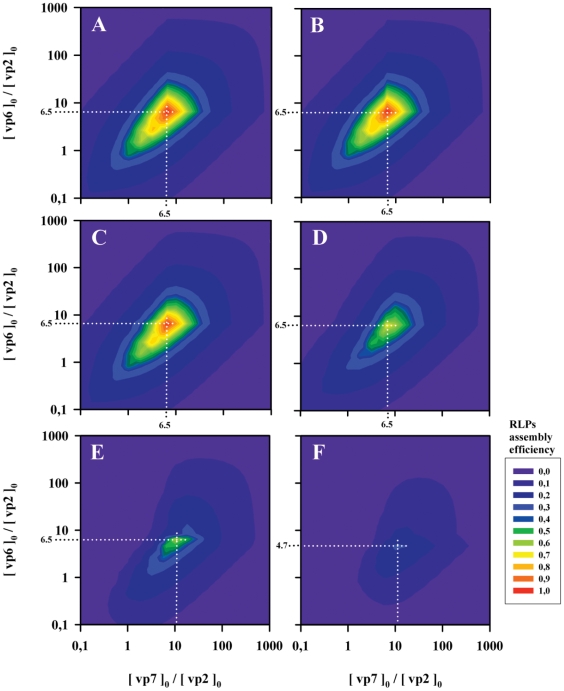
The effect of initial protein concentrations and Gibbs free energy of subunit association on RLP assembly efficiency. The initial concentration of vp2, **[vp2]_0_**, was kept constant at 1 M while the initial concentrations of vp6 and vp7, **[vp6]_0_** and **[vp7]_0_** respectively, varied from 0.1 M to 1000 M. The Gibbs free energies of vp2, vp6 and vp7 subunit association were considered equal (**ΔG^0^_vp2,3-mer_** = **ΔG^0^_vp6,13-mer_** = **ΔG^0^_vp7,13-mer_**). Graph **A** shows the RLP assembly efficiency for the irreversible model while graphs **B** to **F** show the assembly efficiency of the thermodynamic model for **ΔG^0^_vp7,13-mer_** values of −4.08, −2.58, −1.5, −0.75 and −0.408 kcal.mol^−1^, respectively. The RLP assembly efficiency is defined as the ratio between the mass of intact RLP and the mass of initial proteins.

Figure 5a shows the RLP assembly efficiency for the irreversible model while figures 5b–f show the assembly efficiency of the thermodynamic model for **ΔG^0^_vp7,13-mer_** values of −4.08, −2.58, −1.5, −0.75 and −0.408 kcal.mol^−1^, respectively.

As expected, the assembly efficiencies derived from the irreversible model are always higher than those obtained from the equilibrium model. As expected, by decreasing **ΔG^0^_vp7,13-mer_**, the equilibrium model solution converges to the irreversible model solution (Figures 5a and 5b).

The maximum RLP efficiency is obtained for initial proteins concentrations coincident to the particle protein mass composition (vp2∶vp6∶vp7 - 120∶780∶780 molecules). This stems from the fact that RLP being a triple-layered particle, it must have underneath a DLP and DLP a SLP. This optimum point holds for sufficiently low **ΔG^0^_vp7,13-mer_**, namely **ΔG^0^_vp7,13-mer_** = −4.08, −2.58 and −1.5 kcal.mol^−1^. Significant deviations are observed for higher **ΔG^0^_vp7,13-mer_** such as −0.75 and −0.408 kcal.mol^−1^, where the optimal point deviates to (**[vp6]_0_/[vp2]_0_** = 6.5, **[vp7]_0_/[vp2]_0_** = 10.1) and (**[vp6]_0_/[vp2]_0_** = 4.7, **[vp7]_0_/[vp2]_0_** = 10.1) respectively.

This shows that, for low Gibbs free energies, thermodynamics maximizes the formation of intact icosahedra whenever vp2, vp6 and vp7 are provided at the exact RLP mass composition. When Gibbs free energies increase, there is a translocation of proteins' pseudo-critical concentrations and intact icosahedra are no longer maximized when vp2, vp6 and vp7 are provided at the exact RLP mass composition.

It should be noted that the dependency between assembly efficiency and Gibbs free energies of subunits association is highly non-linear. For values of Gibbs free energy below −2 kcal.mol^−1^, assembly efficiency is practically insensitive of Gibbs free energy since the initial proteins concentrations are above the pseudo-critical value, which guarantees that particle formation is maximized. Above this threshold, exponential decay of intact icosahedra to unassembled subunits is observed.

### Main conclusions

In this study, we computationally analysed the effect of thermodynamic equilibrium on the assembly efficiency of a triple-layered rotavirus VLP. To this purpose, Zlotnick's thermodynamic equilibrium framework was applied to an hierarchical assembly model wherein protein layers are sequentially formed from the innermost to the outermost layer following the principle of most stable intermediate structures, which are ultimately the ones favoured by thermodynamics. From the combined *in vitro* and *in silico* analysis, the following main conclusions can be withdrawn from this study:

*In vitro* assembly and disassembly data indicated variations in Gibbs free energy of vp7 subunit association between −2.9 and −4.8 kcal mol^−1^. The Ca^2+^ and Na^+^ ions significantly decrease **ΔG^0^_vp7,13-mer_** thus stabilizing the particle. Proof-of-principle of disassembly followed by reassembly by manipulating physicochemical properties is provided, which supports a thermodynamically controlled assembly;
Also *in vitro* light scattering data evidences a slow and reversible assembly of DLP to RLP, which is untypical of kinetic traps thus further supporting the fidelity of thermodynamically controlled assembly;
For minimising the formation of kinetically trapped products, such as helix- and sheet-like structures of vp6, it is essential to ensure a spatial and/or time controlled assembly. Furthermore, the formation of such kinetically trapped products can be explained on the basis of the thermodynamic assembly under non-stoichiometric conditions and Gibbs free energies of association within those normally observed for virus and protein assembly (−12 to −4 kcal/mol [25]–[26]). Only in extreme conditions, such as very negative Gibbs free energy of association, the dissociation rate becomes negligible with respect to the association rate, and the most likely structures are not the most stable ones (completely formed DLP), thus leading to the accumulation of malformed/kinetically trapped products. Otherwise, the thermodynamic control prevails.
Computational analysis shows that except for harshly unfavourable thermodynamic conditions (**ΔG^0^_vp7,13-mer_**>−0.75 kcal.mol^−1^), the RLP assembly efficiency is maximised and approaches 100% whenever proteins are provided at exact VLP protein mass composition. In such conditions, the thermodynamic and irreversible models are equivalent;
However, small deviations of protein concentrations in relation to the VLP protein mass composition results in a very significant decrease of assembly efficiency eventually far beyond the expected when compared to the irreversible model. This effect is even more pronounced for high **ΔG^0^_vp7,13-mer_** where an exponential decay towards intermediate species is observed closer to the optimal operational point. This clearly points out towards the fact that the formation of complete icosahedral particles is restricted to a narrow range of association energies and protein concentrations.
The assembly efficiency is very much affected by the particle complexity. Not only because a more complex particle has a higher number of stable intermediates all of them potentially accumulating in solution, but mainly because each structural subunit affects the equilibrium of all intermediate species and also of the other structural subunits. To illustrate this fact, for a low **ΔG^0^_vp7,13-mer_** of −1 kcal mol^−1^ and protein concentrations equal to the VLP protein mass composition the assembly efficiency is 27.5% for the complete particle. Under a scenario of 10% vp7 excess, RLP assembly efficiency increases to 59.4% whereas at 10% of vp2 limitation this value decreases to 20.6%.

Taken together this study shows that an equilibrium analysis of complex multi-layered VLPs may provide important insights about the feasibility of *in vivo* production systems. Since the formation of complete icosahedral particles is restricted to a narrow range of association energies and protein concentrations, the choice of host system should take these factors into consideration. Moreover, the dynamic control of intracellular protein expression rates also becomes critical to minimize wasted proteins. Finally, such models find direct application in the design of *in vitro* physicochemical conditions for disassembly-reassembly experiments towards economical VLP production.

## Methods

### Model

The RLP assembly pathway adopted in this study is based on three main hypotheses. The first hypotheses is that vp2 alone self-assembles into icosahedral structures without the need of vp6 or vp7, thus forming the innermost layer and core of RLP. This is in agreement with Caspar and Klug theory that subunits constructed according to principles of efficient design are able to self-assemble without the need of any external organizer [27]. While this is true for vp2 [18], [28]–[29], experimental evidences show that vp6 self-assembles into tubular- or sheet-like structures [30] and vp7 forms soluble, calcium-dependent trimers as main structures [31] rather than spherical/icosahedra structures.

The second hypotheses is that the assembly of vp2 into single-layered icosahedral particles (SLP) proceeds according to a specific, oriented sequence towards the next most stable arrangement until the shell is completely formed. This hierarchical assembly assumption is again supported by Caspar and Klug (1962) theory that subunits assemble in only one way to form the most stable shell [27].

As third hypotheses, we assume spatial and/or time controlled assembly of vp2, vp6 and vp7, following toa template driven assembly process that minimizes the formation of kinetic traps of vp6 and vp7 (the issue of kinetic traps is discussed further below). This translates into a hierarchical RLP assembly pathway with structural subunits of the proceeding layer docking only after the preceding layer is completely formed:

1. Self-assembly of vp2 structural subunits to form icosahedral SLP;
2. Docking of vp6 structural subunits onto completely built SLPs to form DLP;
3. Docking of vp7 structural subunits onto completely built DLPs to form triple-layered vp2/vp6/vp7 RLPs.

In fact, *in vivo* RLP assembly uses either compartmentalisation or delayed expression to take advantage of the maximum efficiency of template assembly. This is supported by previous evidence that the three viral proteins are synthesized in different ribosomes: vp2 and vp6 are synthesized on free ribosomes while vp7 is synthesized on ER-associated ribosomes [32]–[33]. This suggests that DLPs may assemble in the cytoplasm, bud into the endoplasmic reticulum and then acquire the outer vp7 layer [34].

Under these considerations, we have applied the Zoltnick's thermodynamic equilibrium framework (see section “Zlotnick's equilibrium modelling framework” for details) to describe the equilibrium of **n = 60** protein macrostructures comprising both structural subunits and fully formed intermediate icosahedral layers as specified in Tables S1, S2, S3 and S4 and explained in detail below.

### Formation of the vp2 inner core

In the absence of any other protein, recombinant vp2 form SLPs with diameters of about 45 nm and hexagonal outlines while no vp2 monomers or dimmers are observed [18], [28]–[29]. This suggests that the inner RLP layers share the same icosahedral geometry as native rotavirus vp2 which are arranged as 60 dimers on an unusual **T** = 1 icosahedral lattice as determined by cryo-electron microscopy [35]. Based on the structure of native vp2 layer and the assembly pathway defined in the equilibrium model of Zlotnick [2], we have hypothesized that the expressed vp2 first form dimers, which subsequently form stable structural subunits. These subunits constitute the 20 triangular faces of an icosahedron where each subunit consists of 3 dimers. The formation of structural subunits is assumed to be an equilibrium process yet favoring the assembled subunits. The interaction between the three dimers in a subunit occurs at the 3-fold axes whereas the contact between consecutive subunits occurs at the 5-fold axes. These assumptions are in accordance with X-ray crystal structure of the rotavirus inner capsid particle [36] or the bluetongue virus core, which has a similar icosahedral structure [37].

Table S2 lists the assembly intermediates and factors describing the assembly of SLPs. The Gibbs free energy of vp2 subunit association is defined by **ΔG^0^_vp2,3-mer_**. The subunits of SLPs are linked by strong hydrophobic interactions [36], [38], thus **ΔG^0^_vp2,3-mer_**<<0 kcal.mol^−1^.

Modeling parameters for this layer are: **n** = 20 (vp2 layer has icosahedral geometry with 20 structural subunits), **S_2,n_** = 3 (each structural subunit consists of 3 icosahedral asymmetric units) and **z** = 2 (each asymmetric unit is formed by 2 proteins) [2], [39].

The effect of calcium concentration, pH and temperature on the Gibbs free energy of vp2 subunit association was not considered in the model and is open for future studies.

### Formation of the vp6 intermediate layer

Recombinant vp6 rapidly forms oligomers *in vivo*, mostly as trimeric structures. The dimeric form of the protein stabilized by disulfide bonds may exist but represent either a step in the formation of trimers or an abnormal oligomerization while monomers of vp6 are rare [29]. Upon treatment of DLPs with 1.5 M of calcium chloride (CaCl_2_), the outer layer is removed but trimers do not disaggregate. These experimental evidences support vp6 trimers as the building blocks of the intermediate vp6 layer with **T** = 13 quasi-equivalent icosahedral geometry similar to the native rotavirus vp6 [38]. In such an icosahedral lattice, there are five distinct positions for the vp6 trimers on the surface of the vp2 layer. They are designated as **P**, **Q**, **R**, **S**, and **T** [36], similarly to what is observed with vp7 trimers in the bluetongue virus [37], [40]; the precise juxtaposition of these trimers varies slightly in accordance with the requirements of quasi-equivalence. Based on this structure, we have hypothesized that the vp6 layer is formed by 20 structural subunits, each one consisting of 13 vp6 trimers. The assembly of one subunit involves the initial contact of vp6 trimer **T** with the vp2 layer at its icosahedral 3-fold axes (**ΔG^0^_vp6T,SLP_** – Gibbs free energy for this association) and then the addition of trimers **Q**, **R**, **S** and **P** around trimer **T** in a sequential manner, obeying to particle's asymmetry and chirality (Table S3). The assembly sequence detail for the vp6 13-mer (structure **n** = 20.13) scores 1 unit of **ΔG^0^_vp6QRSP,T+SLP_** (**N_c,n_** = 1) for each additional trimer **Q**, **R**, **S** or **P**. This scoring value includes the Gibbs free energy of association between trimers **Q**, **R**, **S** or **P** and trimer **T** – **ΔG^0^_vp6QRSP,T_** – and between trimers **Q**, **R**, **S** or **P** and the vp2 layer – **ΔG^0^_vp6QRSP,SLP_** – thus ending up in
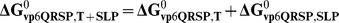
In virtue of model simplification and computation time, the assembly of vp6 structural subunits was lumped together with the Gibbs free energy of vp6 subunit association given by
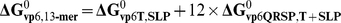
The assembly of vp6 layer results from the addition of structural subunits to the most stable assembly intermediates (Table S4). This order of assembly is consistent with a gradient of trimer association from a single nucleation site as proposed by Limn and Roy (2003) for the construction of the vp7 layer of the bluetongue virus [41].

The X-ray structure of rotavirus vp6 shows that trimer interface at the base contains mostly hydrophilic/negatively charged residues [38]. In addition, vp6 trimers interact with the outer surface of vp2 in the SLP through a network of hydrogen bonds and van der Waals contacts [36]. The interaction between vp6 trimer **T** and SLP is the only one having three contact points and is thus expected to be the most stable interaction among the possible vp6trimer-SLP interactions. The contact between vp6 trimers **P** of adjacent subunits is ensured by strong lateral non-equivalent interactions and occurs across the quasi 2-fold axes but closer to the 3-fold axes and at the icosahedral 5-fold axes [36],[42]. Based on these evidences and on the fact that solubilisation of vp6 layer is only accomplished using chaotropic agents, it is possible to assume for recombinant DLPs that
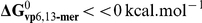

Table S4 lists the assembly intermediates and factors describing the assembly of the second RLP layer. Modeling parameters for this layer are: **n** = 20 (vp6 layer has icosahedral geometry with 20 structural subunits), **S_2,n_** = 13 (there are 13 equivalent orientations for the each trimer in the incoming subunit) and **z** = 3 (each asymmetric unit is formed by 3 proteins) [2], [39]. The concentration of unassociated vp6 subunits at equilibrium (M), **[** ***free*** **vp6]**, and the concentration of vp6 trimers - the building blocks of a vp6 structural subunits - **[vp6_trimers_]**, correlate according to the following equation: **[** ***free*** **vp6]** = **[vp6_trimers_]^13^**.

### Formation of the outer vp7 layer

The structure of the outer rotavirus layer is similar to the vp6 layer: 780 molecules of vp7 arranged as 260 trimers in a **T** = 13 quasi-equivalent icosahedral geometry [34]. In the native virus there are solvent channels that go across the rotavirus structure, through which mRNA transcripts emerge from the viral particle and aqueous material and biochemical substrates are transported into and out of the capsid; these channels persist even after the assembly of the outer layer [42]. This suggests that vp7 trimers interact in a one-to-one fashion with the vp6 trimers in the inner layer by contacting top of one with bottom of the other [38]. Based on these similarities, we have hypothesized that the vp7 layer construction in RLPs obeys to a specific sequence similar to that observed for the vp6 layer (vp7 layer composed by 20 stable subunits of 13 vp7 trimers). In addition, the existence of five distinct positions for the vp7 trimers on the surface of the DLP was considered as in the vp6 case. The solubilisation of the outer layer from the RLP is achieved using low calcium concentration, by the addition of chelating agents such as EDTA or EGTA or by increasing the temperature [21]. No significant effect is observed on SLPs or DLPs. In a first study, Li and co-workers were able to identify two interaction points between vp6 and vp7 trimers [43]. Since these contact points were apparently uncharged, the authors suggested that vp6–vp7 trimer interactions were stabilized by hydrophobic interactions. However, a recent study by Chen and co-workers showed using high-resolution cryo-EM that the outward surface of vp6 trimers and the inward surface of vp7 trimers interact using van der Waals contacts and hydrogen bonds [23]. These evidences suggest that the Gibbs free energy of vp7 subunit association **ΔG^0^_vp7,13-mer_**<<0 kcal.mol^−1^.

As in the case of vp6, the assembly of vp7 structural subunits was lumped together. Hence, the Gibbs free energy of vp7 subunit association can be defined as
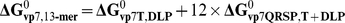
where **ΔG^0^_vp7T,DLP_** is the Gibbs free energy for the association of vp7 trimer **T** with the DLP layer, **ΔG^0^_vp7QRSP,T+DLP_** the Gibbs free energy of association between vp7 trimers **Q**, **R**, **S** or **P** and trimer **T** and between vp7 trimers **Q**, **R**, **S** or **P** and the DLP layer. Table S1 lists the assembly intermediates and factors describing the assembly of vp7 RLP layer, where **[** ***free*** **vp7]** is the concentration of unassociated vp7 subunits at equilibrium (M).

Modeling parameters for this layer are: **n** = 20 (vp7 layer has icosahedral geometry with 20 structural subunits), **S_2,n_** = 13 (there are 13 equivalent orientations for the each trimer in the incoming subunit) and **z** = 3 (each asymmetric unit is formed by 3 proteins) [2], [39].

### Potential impact of kinetic traps on the model

Kinetically trapped intermediate products are observed experimentally *in vivo* and *in vitro* as a direct consequence of inaccurate thermodynamic, temporal and spatial conditions. Zeng and co-workers observed *in vitro* that highly concentrated preparations of recombinant vp2 induce the formation of helix- and sheet-like structures of vp2 [4]. The prevalence of these structures correlates well with the concentration of vp2, i.e. the higher the concentration of vp2, the higher is the probability of generating malformed/kinetically trapped species. Interestingly, by diluting these highly concentrated vp2 preparations, the malformed structures are reconverted into spherical shaped structures, thus demonstrating that kinetic traps are reversible. In another study, malformed/kinetically trapped species such as aggregates of vp7 trimers and particles composed of vp6+vp7 or vp2+vp7 were identified [25]. Although experimentally observed *in vivo* or *in vitro*, these structures are present at very low amounts, are rather heterogeneous and have low stability. The formation of these and other malformed/kinetically trapped products (e.g. helix- and sheet-like structures of vp6 [26]) can be explained on the basis of the thermodynamic assembly under non-stoichiometric conditions and Gibbs free energies of association within those normally observed for virus and protein assembly (−12 to −4 kcal/mol [44]–[45]). For example, if vp6 is at limiting concentrations when compared to SLP, it will be diluted on the available SLP particles and DLP will not be obtained. On the other hand, if vp6 is in excess, trimers of vp6 and tubular-like structures of vp6 will be formed and accumulate once all SLP particles had yielded DLP. Only in extreme conditions such as very negative Gibbs free energy of association, where consequently the dissociation rate is negligible with respect to the association rate, the more probable structures are not the most stable ones (completely formed DLP), thus leading to the accumulation of malformed/kinetically trapped products. Otherwise, the thermodynamic control prevails.

### RLPs production, purification and analysis

The production of RLPs, DLPs and vp7 have been described in detail elsewhere [46]–[47]. Briefly, for RLP production, *Spodoptera frugiperda Sf*-9 cells were infected at a cell concentration of 3×10^6^ cell.ml^−1^ with three monocistronic recombinant baculoviruses enclosing the genes coding for vp2 fused to green fluorescent protein (GFP) (BacRF2A-GFP) [18], vp6 (BacVP6C) [48] and vp7 (BacRF7), all of bovine rotavirus strain RF. The multiplicity of infection (MOI), i.e., number of virus per cell used was 3 (vp2)+3 (vp6)+3 (vp7). For DLP production, *Sf*-9 cells were infected at a cell concentration of 3×10^6^ cell.ml^−1^ with two monocistronic recombinant baculoviruses coding for vp2 fused to GFP (BacRF2A-GFP) and vp6 (BacVP6C); the MOI used was 3 (vp2)+3 (vp6). For vp7 production, *Sf*-9 cells were infected at a cell concentration of 1×10^6^ cell.ml^−1^ with monocistronic recombinant baculoviruses coding for vp7 (BacRF7); the MOI used was 5 virus.cell^−1^. All productions were performed in a 2 L stirred tank bioreactor (Sartorius-Stedim Biotech GmbH, Melsungen, Germany). The glass bioreactor was equipped with two Rushton turbines (standard geometry), a water recirculation jacket to control the temperature and a sparger for gas supply; culture temperature and gas flow were kept constant at 27°C and 0.01 vvm (gas volume per culture volume per minute), respectively. The percentage of dissolved oxygen in the culture was controlled at 30% with the agitation rate (70 to 250 rpm) and the concentration of oxygen in the gas mixture (0 to 100%) (control cascade). The pH was monitored but not controlled since it did not vary significantly throughout the culture time. The abovementioned conditions were previously optimized [49].

The purification of RLPs, DLPs and vp7 was performed using *in-house* developed methods consisting in depth filtration, ultrafiltration, affinity-, size exclusion- and anion exchange-chromatography as stepwise unit operations [22], [47]. The total protein content in purified RLPs, DLPs and vp7 was determined using the BCA protein quantification assay kit (96-well plate protocol) (Pierce, Rockford, US) [46]–[47].

The kinetics of *in vitro* RLP assembly and disassembly was monitored using 90° light scattering whereas the analysis of intact RLPs and DLPs was performed by capillary zone electrophoresis [21].

### In vitro assembly and disassembly assays

As described in [21], *in vitro* RLPs assembly experiments using purified DLP and vp7 were carried out in TNC buffer at pH 5.5, 25°C, NaCl 0.1 M and Ca^2+^ 1 mM. The initial concentrations of DLP and vp7 were 10 pM and 7.8 nM, respectively. The rationale for this choice is providing DLPs and vp7 at concentrations that obey to the RLP stoichiometric composition (vp2∶vp6∶vp7 - 120∶780∶780 molecules). The effect of physicochemical parameters on the Gibbs free energy of vp7 subunit association and assembly efficiency was investigated. Higher pH (8), temperature (35°C), concentration of NaCl (0.5 M) or Ca^2+^ (5 mM) were tested (see table 1).

Once solution reaches equilibrium, assembly efficiency was calculated as follows:
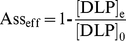
(16)with **[DLP]_0_** the initial concentration of DLP and **[DLP]_e_** the concentration of DLP at equilibrium.

*In vitro* RLP disassembly experiments were performed with chelating agents (EDTA or EGTA at 1 mM and 2 mM in D-PBS and TNC) at 35°C. The composition of D-PBS and TNC buffers was as follows: D-PBS (137 mM NaCl, pH 7.4, 0.9 mM CaCl_2_ and 0.493 mM magnesium chloride (MgCl_2_)); TNC (20 mM Tris-HCl, 100 mM NaCl, pH 7.3 and 0.9 mM CaCl_2_). The initial concentration of RLP was 7 nM.

The disassembly efficiency was calculated as follows:
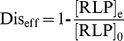
(17)with **[RLP]_0_** the initial concentration of RLP and **[RLP]_e_** the concentration of RLP at equilibrium.

The kinetics of *in vitro* RLP disassembly was analyzed for an initial RLP concentration of 1 nM, 25°C and two conditions: 1 mM of EDTA in TNC and 1 mM of EGTA in D-PBS.

The reassembly of the particles previously disassembled with EDTA or EGTA 2 mM was studied using dialysis; the conditions used for the reassembly were 5 mM of Ca^2+^ and 25°C.

The data collected in these experiments allowed estimating the Gibbs free energies of vp7 subunit association to DLP by fitting the model described next.

### Zlotnick's equilibrium modelling framework

The self-assembly of a generic polyhedral virus can be described mathematically using the equilibrium theory proposed by Zlotnick in 1994. This modelling approach is based on the following principles:

- Structural subunits are very stable protein aggregates forming the building blocks in the assembly process;
- Structural subunits are sequentially added to the most stable intermediate until a complete layer is formed (see Table S1, S2 and S4);
- Only the most stable conformation, i.e. the arrangement with the greatest number of inter-subunit contacts, is considered;
- Assembly reactions are rapid, reaching equilibrium in milliseconds;
- Only ideal icosahedral geometry is allowed for each intermediate;
- Nucleation and cooperativity are neglected – the light scattering data here presented corroborated the inexistence of kinetic traps and as such kinetic limiting steps were not consider;
- The contact between adjacent subunits results in a characteristic Gibbs free energy variation, **ΔG^0^_n_**;
- All interactions between identical subunits have the same characteristic **ΔG^0^_n_** Gibbs free energy, irrespective of the particle size.

The assembly of structural subunits into a complete layer involves a series of equilibrium reactions, statistical components and thermodynamic factors. The association constant, **K_n_**, of a species **n**, a specific arrangement of **m** structural subunits, can be uncoupled into statistical components **S_1,n_** and **S_2,n_**, and a non-statistical association constant **K′_n_**:
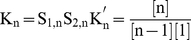
(18)The structural subunit **m** = **n** = **1** is defined as the stable building block and **n−1** is defined as the intermediate composed of **m−1** subunits. **S_1,n_** is defined as the ratio between two factors: the number of ways of forming a species **n** (from the preceding most stable intermediate, **n−1**, and free subunit **n** = **1** – **Build-up**) and the number of ways of dissociating the intermediate species **n** into the respective reactants, **n−1** (**Build-down**). **S_2,n_** describes the degeneracy of the incoming subunit in species **n**; this statistical factor depends on the number of equivalent orientations that each incoming icosahedral asymmetric unit, called oligomer (cluster of proteins – the fundamental of the assembly reaction), adopts in a structural subunit **m**. The non-statistical association constant **K′_n_** is a function of the number of contacts between subunits for each newly formed species **n**, **N_c,n_**, and **ΔG^0^_n_**, and does not include the degeneracy of the interaction:
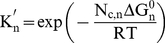
(19)with **R** = 1.987 cal.mol^−1^ K^−1^ the ideal gas constant and **T** the temperature (K). The concentration of each intermediate species **n** can be estimated by rearranging Eq. 3 and Eq. 4, so that:
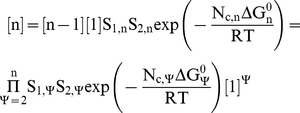
(20)The concentration of each species is a function of the stable building block, species **1**, and **ΔG^0^_n_**. The total protein in solution at time **t**, **Prot (t)**, can be calculated from the species concentration **1** to **n**:
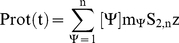
(21)where **m_Ψ_** represents the number of structural subunits in species **ψ** = **n** and **z** the number of proteins in each oligomer.

## Supporting Information

Table S1 Assembly intermediates and factors describing the formation of single-layered vp2 particles (SLP).
(DOC)

Table S2 Assembly intermediates and factors describing the formation of the first vp6 structural subunit, from the interaction between vp6 trimers (building blocks), on top of SLP.
(DOC)

Table S3 Assembly intermediates and factors describing the formation of the vp6 layer, from 20 vp6 structural subunits, on top of SLP.
(DOC)

Table S4 Assembly intermediates and factors describing the formation of the vp7 layer, from 20 vp7 structural subunits, on top of DLP.
(DOC)
