# Supplementary material for: On the Effect of Thermodynamic Equilibrium on the Assembly Efficiency of Complex Multi-Layered Virus-Like Particles (VLP): the Case of Rotavirus VLP
Source: PLoS Comput Biol. 2012 Feb 16;8(2):e1002367. doi: 10.1371/journal.pcbi.1002367 (PMC3280969; doi:10.1371/journal.pcbi.1002367)
Supplement: Table S1 — Assembly intermediates and factors describing the formation of single-layered vp2 particles (SLP). (DOC) [file pcbi.1002367.s001.doc]

**Table S1.** Assembly intermediates and factors describing the formation of the vp7 layer, from 20 vp7 structural subunits, on top of DLP.

| **n** | **Model** | **Build** | | **S_1,n_** | **N_c,n_** | **[n]** |  | **n** | **Model** | **Build** | | **S_1,n_** | **N_c,n_** | **[n]** |
| --- | --- | --- | --- | --- | --- | --- | --- | --- | --- | --- | --- | --- | --- | --- |
|  |  | **up** | **down** |  |  |  |  |  |  | **up** | **down** |  |  |  |
| 40 |  | DLPs | | | | |  | 51 |  | 6 | 1 | 6/1 | 2 | $\left[40\right]\times13^{11}\times240\times e^{\left(-\frac{24\times\Delta G_{vp7,13-mer}^{0}}{RT}\right)}\left[free\text{ vp7}\right]^{11}$ |
| 41 |  | 20 | 1 | 20/1 | 1 | $\left[40\right]\times13\times20\times e^{\left(-\frac{1\times\Delta G_{vp7,13-mer}^{0}}{RT}\right)}\left[free\text{ vp7}\right]$ |  | 52 |  | 1 | 4 | 1/4 | 3 | $\left[40\right]\times13^{12}\times60\times e^{\left(-\frac{27\times\Delta G_{vp7,13-mer}^{0}}{RT}\right)}\left[free\text{ vp7}\right]^{12}$ |
| 42 |  | 3 | 1 | 3/1 | 2 | $\left[40\right]\times13^{2}\times60\times e^{\left(-\frac{3\times\Delta G_{vp7,13-mer}^{0}}{RT}\right)}\left[free\text{ vp7}\right]^{2}$ |  | 53 |  | 2 | 1 | 2/1 | 2 | $\left[40\right]\times13^{13}\times120\times e^{\left(-\frac{29\times\Delta G_{vp7,13-mer}^{0}}{RT}\right)}\left[free\text{ vp7}\right]^{13}$ |
| 43 |  | 4 | 2 | 4/2 | 2 | $\left[40\right]\times13^{3}\times120\times e^{\left(-\frac{5\times\Delta G_{vp7,13-mer}^{0}}{RT}\right)}\left[free\text{ vp7}\right]^{3}$ |  | 54 |  | 2 | 2 | 2/2 | 3 | $\left[40\right]\times13^{14}\times120\times e^{\left(-\frac{32\times\Delta G_{vp7,13-mer}^{0}}{RT}\right)}\left[free\text{ vp7}\right]^{14}$ |
| 44 |  | 2 | 2 | 2/2 | 2 | $\left[40\right]\times13^{4}\times120\times e^{\left(-\frac{7\times\Delta G_{vp7,13-mer}^{0}}{RT}\right)}\left[free\text{ vp7}\right]^{4}$ |  | 55 |  | 1 | 5 | 1/5 | 3 | $\left[40\right]\times13^{15}\times24\times e^{\left(-\frac{35\times\Delta G_{vp7,13-mer}^{0}}{RT}\right)}\left[free\text{ vp7}\right]^{15}$ |
| 45 |  | 1 | 5 | 1/5 | 3 | $\left[40\right]\times13^{5}\times24\times e^{\left(-\frac{10\times\Delta G_{vp7,13-mer}^{0}}{RT}\right)}\left[free\text{ vp7}\right]^{5}$ |  | 56 |  | 5 | 1 | 5/1 | 2 | $\left[40\right]\times13^{16}\times120\times e^{\left(-\frac{37\times\Delta G_{vp7,13-mer}^{0}}{RT}\right)}\left[free\text{ vp7}\right]^{16}$ |
| 46 |  | 5 | 1 | 5/1 | 2 | $\left[40\right]\times13^{6}\times120\times e^{\left(-\frac{12\times\Delta G_{vp7,13-mer}^{0}}{RT}\right)}\left[free\text{ vp7}\right]^{6}$ |  | 57 |  | 2 | 2 | 2/2 | 3 | $\left[40\right]\times13^{17}\times120\times e^{\left(-\frac{40\times\Delta G_{vp7,13-mer}^{0}}{RT}\right)}\left[free\text{ vp7}\right]^{17}$ |
| 47 |  | 2 | 2 | 2/2 | 2 | $\left[40\right]\times13^{7}\times120\times e^{\left(-\frac{14\times\Delta G_{vp7,13-mer}^{0}}{RT}\right)}\left[free\text{ vp7}\right]^{7}$ |  | 58 |  | 2 | 4 | 2/4 | 3 | $\left[40\right]\times13^{18}\times60\times e^{\left(-\frac{43\times\Delta G_{vp7,13-mer}^{0}}{RT}\right)}\left[free\text{ vp7}\right]^{18}$ |
| 48 |  | 1 | 2 | 1/2 | 3 | $\left[40\right]\times13^{8}\times60\times e^{\left(-\frac{17\times\Delta G_{vp7,13-mer}^{0}}{RT}\right)}\left[free\text{ vp7}\right]^{8}$ |  | 59 |  | 2 | 3 | 2/3 | 3 | $\left[40\right]\times13^{19}\times40\times e^{\left(-\frac{46\times\Delta G_{vp7,13-mer}^{0}}{RT}\right)}\left[free\text{ vp7}\right]^{19}$ |
| 49 |  | 4 | 1 | 4/1 | 2 | $\left[40\right]\times13^{9}\times240\times e^{\left(-\frac{19\times\Delta G_{vp7,13-mer}^{0}}{RT}\right)}\left[free\text{ vp7}\right]^{9}$ |  | 60 |  | 1 | 20 | 1/20 | 4 | $\left[40\right]\times13^{20}\times2\times e^{\left(-\frac{50\times\Delta G_{vp7,13-mer}^{0}}{RT}\right)}\left[free\text{ vp7}\right]^{20}$ |
| 50 |  | 1 | 6 | 1/6 | 3 | $\left[40\right]\times13^{10}\times40\times e^{\left(-\frac{22\times\Delta G_{vp7,13-mer}^{0}}{RT}\right)}\left[free\text{ vp7}\right]^{10}$ |  |  |  |  |  |  |  |  |
