# Supplementary material for: On the Effect of Thermodynamic Equilibrium on the Assembly Efficiency of Complex Multi-Layered Virus-Like Particles (VLP): the Case of Rotavirus VLP
Source: PLoS Comput Biol. 2012 Feb 16;8(2):e1002367. doi: 10.1371/journal.pcbi.1002367 (PMC3280969; doi:10.1371/journal.pcbi.1002367)
Supplement: Table S2 — Assembly intermediates and factors describing the formation of the first vp6 structural subunit, from the interaction between vp6 trimers (building blocks), on top of SLP. (DOC) [file pcbi.1002367.s002.doc]

**Table S2.** Assembly intermediates and factors describing the formation of single-layered vp2 particles (SLP).

| **n** | **Model** | **Build** | | **S_1,n_** | **N_c,n_** | **[n]** |  | **n** | **Model** | **Build** | | **S_1,n_** | **N_c,n_** | **[n]** |
| --- | --- | --- | --- | --- | --- | --- | --- | --- | --- | --- | --- | --- | --- | --- |
|  |  | **up** | **down** |  |  |  |  |  |  | **up** | **down** |  |  |  |
| 1 |  | - | - | - | - | [1] |  | 11 |  | 6 | 1 | 6/1 | 1 | $3^{10}\times6\times e^{\left(-\frac{13\times\Delta G_{vp2,3-mer}^{0}}{RT}\right)}\left[1\right]^{11}$ |
| 2 |  | 3/2^a^ | 1 | 3/2 | 1 | $3\times\frac{3}{2}\times e^{\left(-\frac{1\times\Delta G_{vp2,3-mer}^{0}}{RT}\right)}\left[1\right]^{2}$ |  | 12 |  | 1 | 4 | 1/4 | 2 | $3^{11}\times\frac{3}{2}\times e^{\left(-\frac{15\times\Delta G_{vp2,3-mer}^{0}}{RT}\right)}\left[1\right]^{12}$ |
| 3 |  | 4 | 2 | 4/2 | 1 | $3^{2}\times3\times e^{\left(-\frac{2\times\Delta G_{vp2,3-mer}^{0}}{RT}\right)}\left[1\right]^{3}$ |  | 13 |  | 2 | 1 | 2/1 | 1 | $3^{12}\times3\times e^{\left(-\frac{16\times\Delta G_{vp2,3-mer}^{0}}{RT}\right)}\left[1\right]^{13}$ |
| 4 |  | 2 | 2 | 2/2 | 1 | $3^{3}\times3\times e^{\left(-\frac{3\times\Delta G_{vp2,3-mer}^{0}}{RT}\right)}\left[1\right]^{4}$ |  | 14 |  | 2 | 2 | 2/2 | 2 | $3^{13}\times3\times e^{\left(-\frac{18\times\Delta G_{vp2,3-mer}^{0}}{RT}\right)}\left[1\right]^{14}$ |
| 5 |  | 1 | 5 | 1/5 | 2 | $3^{4}\times\frac{3}{5}\times e^{\left(-\frac{5\times\Delta G_{vp2,3-mer}^{0}}{RT}\right)}\left[1\right]^{5}$ |  | 15 |  | 1 | 5 | 1/5 | 2 | $3^{14}\times\frac{3}{5}\times e^{\left(-\frac{20\times\Delta G_{vp2,3-mer}^{0}}{RT}\right)}\left[1\right]^{15}$ |
| 6 |  | 5 | 1 | 5/1 | 1 | $3^{5}\times3\times e^{\left(-\frac{6\times\Delta G_{vp2,3-mer}^{0}}{RT}\right)}\left[1\right]^{6}$ |  | 16 |  | 5 | 1 | 5/1 | 1 | $3^{15}\times3\times e^{\left(-\frac{21\times\Delta G_{vp2,3-mer}^{0}}{RT}\right)}\left[1\right]^{16}$ |
| 7 |  | 2 | 2 | 2/2 | 1 | $3^{6}\times3\times e^{\left(-\frac{7\times\Delta G_{vp2,3-mer}^{0}}{RT}\right)}\left[1\right]^{7}$ |  | 17 |  | 2 | 2 | 2/2 | 2 | $3^{16}\times3\times e^{\left(-\frac{23\times\Delta G_{vp2,3-mer}^{0}}{RT}\right)}\left[1\right]^{17}$ |
| 8 |  | 1 | 2 | 1/2 | 2 | $3^{7}\times\frac{3}{2}\times e^{\left(-\frac{9\times\Delta G_{vp2,3-mer}^{0}}{RT}\right)}\left[1\right]^{8}$ |  | 18 |  | 2 | 4 | 2/4 | 2 | $3^{17}\times\frac{3}{2}\times e^{\left(-\frac{25\times\Delta G_{vp2,3-mer}^{0}}{RT}\right)}\left[1\right]^{18}$ |
| 9 |  | 4 | 1 | 4/1 | 1 | $3^{8}\times6\times e^{\left(-\frac{10\times\Delta G_{vp2,3-mer}^{0}}{RT}\right)}\left[1\right]^{9}$ |  | 19 |  | 2 | 3 | 2/3 | 2 | $3^{18}\times1\times e^{\left(-\frac{27\times\Delta G_{vp2,3-mer}^{0}}{RT}\right)}\left[1\right]^{19}$ |
| 10 |  | 1 | 6 | 1/6 | 2 | $3^{9}\times1\times e^{\left(-\frac{12\times\Delta G_{vp2,3-mer}^{0}}{RT}\right)}\left[1\right]^{10}$ |  | 20 |  | 1 | 20 | 1/20 | 3 | $3^{19}\times\frac{1}{20}\times e^{\left(-\frac{30\times\Delta G_{vp2,3-mer}^{0}}{RT}\right)}\left[1\right]^{20}$ |
| ^a^ The build-up factor 3/2, instead of the logically factor 3, is due to the degeneracy of the asymmetric unit. | | | | | | | | | | | | | | |
