# Supplementary material for: On the Effect of Thermodynamic Equilibrium on the Assembly Efficiency of Complex Multi-Layered Virus-Like Particles (VLP): the Case of Rotavirus VLP
Source: PLoS Comput Biol. 2012 Feb 16;8(2):e1002367. doi: 10.1371/journal.pcbi.1002367 (PMC3280969; doi:10.1371/journal.pcbi.1002367)
Supplement: Table S3 — Assembly intermediates and factors describing the formation of the vp6 layer, from 20 vp6 structural subunits, on top of SLP. (DOC) [file pcbi.1002367.s003.doc]

**Table S3.** Assembly intermediates and factors describing the formation of the first vp6 structural subunit, from the interaction between vp6 trimers (building blocks), on top of SLP.

| **n** | **Model** | **Build** | | **S_1,n_** | **N_c,n_** | **[n]** |
| --- | --- | --- | --- | --- | --- | --- |
|  |  | **Up** | **down** |  |  |  |
| 20.1 | 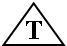 | 20 | 1 | 20/1 | 1 | $\left[20\right]\times20\times e^{\left(-\frac{1\times\Delta G_{vp6T,SLP}^{0}}{RT}\right)}\left[\text{vp6}_{\text{trimers}}\right]$ |
| 20.2 | 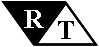 | 3 | 1 | 3/1 | 1 | $\left[20\right]\times60\times e^{\left(-\frac{1\times\Delta G_{vp6T,SLP}^{0}+1\times\Delta G_{vp6QRSP,T+SLP}^{0}}{RT}\right)}\left[\text{vp6}_{\text{trimers}}\right]^{2}$ |
| 20.3 | 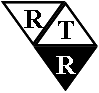 | 2 | 2 | 2/2 | 1 | $\left[20\right]\times60\times e^{\left(-\frac{1\times\Delta G_{vp6T,SLP}^{0}+2\times\Delta G_{vp6QRSP,T+SLP}^{0}}{RT}\right)}\left[\text{vp6}_{\text{trimers}}\right]^{3}$ |
| 20.4 | 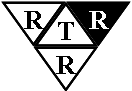 | 1 | 3 | 1/3 | 1 | $\left[20\right]\times20\times e^{\left(-\frac{1\times\Delta G_{vp6T,SLP}^{0}+3\times\Delta G_{vp6QRSP,T+SLP}^{0}}{RT}\right)}\left[\text{vp6}_{\text{trimers}}\right]^{4}$ |
| 20.5 | 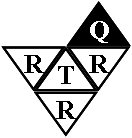 | 3 | 1 | 3/1 | 1 | $\left[20\right]\times60\times e^{\left(-\frac{1\times\Delta G_{vp6T,SLP}^{0}+4\times\Delta G_{vp6QRSP,T+SLP}^{0}}{RT}\right)}\left[\text{vp6}_{\text{trimers}}\right]^{5}$ |
| 20.6 | 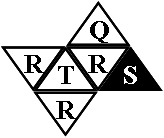 | 3 | 2 | 3/2 | 1 | $\left[20\right]\times90\times e^{\left(-\frac{1\times\Delta G_{vp6T,SLP}^{0}+5\times\Delta G_{vp6QRSP,T+SLP}^{0}}{RT}\right)}\left[\text{vp6}_{\text{trimers}}\right]^{6}$ |
| 20.7 | 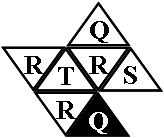 | 2 | 3 | 2/3 | 1 | $\left[20\right]\times60\times e^{\left(-\frac{1\times\Delta G_{vp6T,SLP}^{0}+6\times\Delta G_{vp6QRSP,T+SLP}^{0}}{RT}\right)}\left[\text{vp6}_{\text{trimers}}\right]^{7}$ |
| 20.8 | 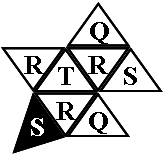 | 2 | 4 | 2/4 | 1 | $\left[20\right]\times30\times e^{\left(-\frac{1\times\Delta G_{vp6T,SLP}^{0}+7\times\Delta G_{vp6QRSP,T+SLP}^{0}}{RT}\right)}\left[\text{vp6}_{\text{trimers}}\right]^{8}$ |
| 20.9 | 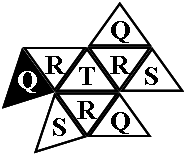 | 1 | 5 | 1/5 | 1 | $\left[20\right]\times6\times e^{\left(-\frac{1\times\Delta G_{vp6T,SLP}^{0}+8\times\Delta G_{vp6QRSP,T+SLP}^{0}}{RT}\right)}\left[\text{vp6}_{\text{trimers}}\right]^{9}$ |
| 20.10 | 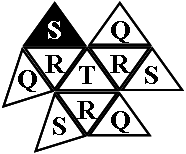 | 1 | 6 | 1/6 | 1 | $\left[20\right]\times1\times e^{\left(-\frac{1\times\Delta G_{vp6T,SLP}^{0}+9\times\Delta G_{vp6QRSP,T+SLP}^{0}}{RT}\right)}\left[\text{vp6}_{\text{trimers}}\right]^{10}$ |
| 20.11 | 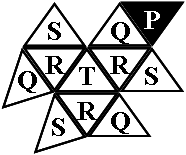 | 3 | 1 | 3/1 | 1 | $\left[20\right]\times3\times e^{\left(-\frac{1\times\Delta G_{vp6T,SLP}^{0}+10\times\Delta G_{vp6QRSP,T+SLP}^{0}}{RT}\right)}\left[\text{vp6}_{\text{trimers}}\right]^{11}$ |
| 20.12 | 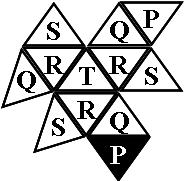 | 2 | 2 | 2/2 | 1 | $\left[20\right]\times3\times e^{\left(-\frac{1\times\Delta G_{vp6T,SLP}^{0}+11\times\Delta G_{vp6QRSP,T+SLP}^{0}}{RT}\right)}\left[\text{vp6}_{\text{trimers}}\right]^{12}$ |
| 20.13 | 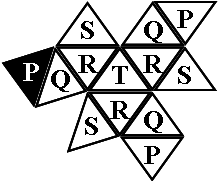 | 1 | 3 | 1/3 | 1 | $\left[20\right]\times1\times e^{\left(-\frac{1\times\Delta G_{vp6T,SLP}^{0}+12\times\Delta G_{vp6QRSP,T+SLP}^{0}}{RT}\right)}\left[\text{vp6}_{\text{trimers}}\right]^{13}$ |
